# Quality of life of physicians in the state of Minas Gerais, Brazil

**DOI:** 10.47626/1679-4435-2021-730

**Published:** 2023-02-03

**Authors:** Walneia Cristina de Almeida Moreira, Fabiola Tatiana de-Souza, Elizabeth Costa Dias, Sarah Ananda Gomes, Marconi Gomes da-Silva, Ana Cláudia Queiroz Gomes, Maria Mercedes Zucheratto Castro

**Affiliations:** 1 Diretoria de Saúde do Trabalhador, Sindicado dos Médicos de Minas Gerais (Sinmed-MG), Belo Horizonte, MG, Brazil.; 2 Diretoria de Educação Continuada, Associação Mineira de Medicina do Trabalho, Belo Horizonte, MG, Brazil.; 3 Academia Mineira de Medicina, Belo Horizonte, MG, Brazil.; 4 Sociedade de Tanatologia e Cuidado Paliativo de Minas Gerais, Belo Horizonte, MG, Brazil.; 5 Diretoria de Promoções Culturais, Associação Médica de Minas Gerais, Belo Horizonte, MG, Brazil.; 6 Conselho Fiscal, Sinmed-MG, Belo Horizonte, MG, Brazil.; 7 Diretoria de Saúde Suplementar, Sinmed-MG, Belo Horizonte, MG, Brazil.

**Keywords:** occupational health, physicians, health promotion, quality of life, saúde do trabalhador, médicos, promoção da saúde, qualidade de vida

## Abstract

**Introduction:**

Studies conducted with physicians from different locations and age groups show a tendency to mental illness and low quality of life in this population.

**Objectives:**

To describe the socioeconomic and quality-of-life profile of medical doctors in the state of Minas Gerais, Brazil.

**Methods:**

Cross-sectional study. A socioeconomic and quality-of-life questionnaire (World Health Organization Quality of Life instrument-Abbreviated version) was applied in a representative sample of physicians working in the state of Minas Gerais. Non-parametric analyses were used to assess outcomes.

**Results:**

The sample was composed of 1,281 physicians, with a mean age of 43.7 years (SD, 11.46) and time since graduation of 18.9 years (SD, 12.1); 12.46% were medical residents, of which 32.7% were in the first year of training. Of the total number of physicians, 66.4% reported feeling overwhelmed and 70.7% were satisfed with their profession. The rate of diagnoses related to depression and anxiety was higher than in the general population. Mean World Health Organization Quality of Life instrument-Abbreviated version score was 60.44±21.72. The analysis of the reported quality-of-life scores showed that younger physicians, especially women, in the first year of residence, with lower income or salary ranges, high workload, and no regular time of obtained lower scores, as well as those who reported diagnoses of depression and/or anxiety.

**Conclusions:**

Some socioeconomic factors may influence the quality of life of the study population. Further studies are needed to develop effective social support and health protection actions for these workers.

## Abstract (pt)

**Introdução:**

Estudos realizados entre profissionais médicos de diferentes localidades e faixas etárias demonstram uma tendência ao adoecimento mental e à baixa qualidade de vida nessa população.

**Objetivos:**

Desenhar o perfil de qualidade de vida de médicos atuantes no estado de Minas Gerais, Brasil.

**Métodos:**

Tratou-se de um estudo transversal. Foram utilizados questionários socioeconômico e de qualidade de vida (WHOQOL-Bref, do inglês World Health Organization Quality of Life Instrument-Abbreviated Version) em amostra representativa de médicos atuantes no estado de Minas Gerais, com utilização de análises não paramétricas na avaliação dos desfechos.

**Resultados:**

A amostra foi composta de 1.281 médicos, com média de idade de 43,7 anos ± desvio-padrão 11,46 e tempo de formado de 18,9 anos ± 12,1; 12,46% estavam cursando residência médica, entre os quais 32,7% no primeiro ano de treinamento. Do total dos médicos, 66,4% se sentiam sobrecarregados e 70,7% se sentiam satisfeitos com a profissão. Observou-se elevado índice de diagnósticos relacionados à depressão e ansiedade, superior ao encontrado na população geral. A média de pontuação do WHOQOL-Bref foi de 60,44 ± 21,72. A análise das pontuações de qualidade de vida mostrou que os médicos mais jovens, em especial do sexo feminino, em seu primeiro ano de residência, que apresentam renda ou faixas salariais menores, com elevada carga horária de trabalho e sem folgas regulares apresentam pontuação inferior, bem como aqueles que informaram diagnósticos de depressão e/ou ansiedade.

**Conclusões:**

Existem fatores socioeconômicos que influenciam positiva ou negativamente na qualidade de vida da população estudada. O aprofundamento nos estudos sobre essa realidade é essencial para o desenvolvimento de ações efetivas e abrangentes de suporte social e de proteção à saúde desses trabalhadores.

## INTRODUCTION

According to a study conducted by the United Nations (UN), in 2019 Brazil ranked fourth among the countries where life expectancy afer 60 years is growing the most. The indicator went from 76 years in 1980 to 82 years in 2019, a 37%-increase, second only to Bolivia, the Republic of Maldives, and South Korea.^1^

In contrast to these findings, a survey conducted by the Regional Council of Medicine of the State of São Paulo (CREMESP) indicates that the survival of physicians is shorter than the life expectancy of the general population, being 69.1 years among men and 59.2 years among women. These findings are surprising, especially regarding female physicians, who have a life expectancy 20 years shorter than the current national projection, and contradict demographic studies that show that women have always lived longer than men in similar social situations.^2^

According to the authors of the study by CREMESP, among the most frequent causes of mortality are cardiovascular diseases, cancer, and diseases of the respiratory system. Suicide mortality, although it represents a small fraction of the events, presents an alarming rate of 3.5 cases per 10,000 physicians when compared to the rate observed in the general population, 3.8 cases per 100,000 inhabitants. The number of suicides is about four times higher among men in the general population, but among physicians, women commit suicide more ofen.^2^

Despite methodological questions about the accuracy of these findings, these results corroborate findings in the technical-scientific literature that point to high rates of depression, anxiety, alcoholism, substance abuse, stress, burnout syndrome, and suicide among physicians.^2,4,5^

Among the explanations for this illness profile of medical workers and the reduction in their life expectancy, the following are pointed out: work overload, inadequate working conditions and infrastructure, multiple contracts, long working hours, intensification and precariousness of labor contracts, and the sharp reduction in medical remuneration in recent years. These factors are reinforced and/or associated with the burden of responsibility inherent to the profession and the massive incorporation of technologies, which are not always available for medical practice.^3^

The growth of illness and mental sufering among physicians in all age groups has shown diferences only in motivation and diagnosis, with a higher level of stress observed in professionals with less than five years of training, possibly related to instability and professional insecurity.^6^

A syndromic condition called ‘house officer stress syndrome’ was described among medical residents, characterized by episodic cognitive disturbances, chronic anger, skepticism, family conflict, depression, suicidal ideation, suicide, and drug abuse.^5^ Carvalho et al.^5^ call atention to the fact that when comparing the situation between medical residents and other professionals, only physicians link work to sufering. The authors also identified higher rates of depression and anxiety in the group of medical residents when compared to the general population, other professionals, and other physicians.^5^

A greater tendency to develop burnout syndrome was identified in the group of more experienced professionals between 40 and 55 years of age. This syndrome is characterized by emotional exhaustion as a central factor and is manifested by feelings of lack of energy, depersonalization or insensitivity towards the patient, family members, and friends, as well as lack of personal fulfillment with a feeling of incompetence. This condition has been associated with the development of depression, decreased productivity, absenteeism, alcohol and drug abuse, and impairment of family and social relationships.^4^

This panorama points to the need to develop health promotion programs for these workers, identifying their profile regarding interaction with work and quality of life, as well as raising awareness and bringing together these professionals to facilitate the discussion on this topic, leading them to seek mutual support and developing actions that promote an improvement in the situation of satisfaction and well-being.

The sense of well-being encompasses social, cultural, and contextual aspects. Conceptually, it can be divided into existential, emotional, personal, and subjective aspects and reflects the individual’s perception of satisfaction with life itself. It is also defined as the study of happiness and is the result of the overall positive balance of afective experiences, leading to an experience of self-acceptance, positive interactions with others, and autonomy.^7,8^

It is common to use the concepts of well-being and quality of life as synonyms since these elements complement each other. However, according to the World Health Organization (WHO), based on the results of a study group on quality of life, it can be conceptualized as an individual’s “perception of his or her position in life in the context of the culture and value system in which he or she lives and concerning his or her goals, expectations, standards, and concerns.”^9,10^

Thus, the components of quality of life include personal well-being linked to the environment, how the individual adjusts to his or her context, and the tools available for development, referring to achieving goals and aspirations.^10^

In this scenario, the institutions representing physicians in the state of Minas Gerais, Brazil - Minas Gerais Medical Association (Associação Médica de Minas Gerais – AMMG), the Regional Medical Council of Minas Gerais (Conselho Regional de Medicina de Minas Gerais - CRMMG), and the Medical Doctors Union (Sindicato dos Médicos de Minas Gerais - SINMEDMG), with the support of the Minas Gerais Academy of Medicine (Academia Mineira de Medicina - AMM) - proposed to conduct a study on the profile of professionals, their interaction with work, and their perception of quality of life, to support the development of health promotion and awareness actions on the subject, seeking to facilitate the meeting and reflection on living and working conditions, and the search for support to enhance well-being.

## METHODS

This was a cross-sectional study. The sample calculation was based on the population of 55,000 active physicians in the state of Minas Gerais in October 2019, considering a confdence interval of 95% and margin of error of 2%. The initial sample was 2,301 professionals.

The instrument used for data collection was a questionnaire with two sections. The first section contains questions about the socioeconomic profile and social interaction of the population, and the second section is for the identification of the quality-of-life profile, using the World Health Organization Quality of Life Instrument – Abbreviated Version (WHOQOL-Bref), version validated in Portuguese and with a score scale ranging from 0 to 100. The score value is presented as the result of the formula: [(Domain-4) x (100/16)].

The application of the questionnaires occurred through the Google Forms platform from October 2019 to January 2020, and before accessing the questions, the participant had to accept and sign the Informed Consent Form (ICF).

The start of data collection was preceded by intensive dissemination on the digital channels of the participating entities and via WhatsApp. An initial return of 1,290 responses was obtained.

The exclusion criteria adopted were not having completed medical school, not working in the state of Minas Gerais, not currently practicing medicine, or not having completed the ICF. Afer the application of the criteria, a sample of 1,281 participants remained.

For analysis of the results, Python and SPSS sofware were used and the Kolmogorov-Smirnov and Shapiro-Wilk normality tests were applied per domain of the WHOQOL-Bref, identifying that the sample rejects the null hypothesis of normality. Therefore, non-parametric tests were adopted in the analysis.

For the binary variables (all with a yes/no format and gender), the Mann-Whitney test was applied to verify whether the groups have equal or different means. For all variables in which the null hypothesis was rejected, we can state that the average quality score difers between the two groups. The p-value adopted was 0.01.

The Kruskal-Wallis test was applied to test the equality between the means of the groups formed by the variables with more than two values. When the null hypothesis is rejected, we can say that the mean of at least two of the groups are different from the others. The p-value adopted was 0.01.

To verify the existence of a significant relationship between the ordinal and continuous variables, Spearman’s correlation was used. The p-value used was 0.01. Thus, we can say that the correlation between the two groups is significant for the coefficients in which the null hypothesis was rejected. Furthermore, it was possible to measure the intensity of this correlation. For values close to zero, we can say that the relationship is weak; for values close to 1 or -1, the relationship is considered strong. Positive values indicate a positive relationship: the higher the value of the variable, the higher the quality-of-life index. However, negative values indicate an inverse relationship: the higher the variable’s value, the lower the quality-of-life index.

About the consistency of the results of the WHOQOL-Bref/SPSS application, Cronbach’s alpha coefficient was applied to confirm the internal consistency of the WHOQOL-Bref in the sample. As all values obtained are between 0.7 and 0.95, we can conclude that instrument’s reliability is good and desirable (Table 1).

**Table 1:** Analysis of the World Health Organization Quality of Life instrument-Abbreviated version (WHOQOL-Bref) by Cronbach’s alpha coefficient

	Global	Psychological	Physical	Environment	Social
Cronbach’s alpha	0.937	0.844922	0.819628	0.817	0.774

The research project was submited to *Plataforma Brasil* and was approved by the Research Ethics Commitee under number 22336719.2.0000.5134.

## RESULTS

The responses of the 1,281 participants were analyzed considering the following variables: demographic profile; professional practice location, social and community interaction profile; and physical and mental health profile.

About the demographic profile of the participants, the mean age was 43.7 years [standard deviation (SD), 11.46], with a mean time of professional experience of 18.9 years (SD, 12.09) and a predominance of women (58.7%); one participant did not indicate sex. From the total, 12.4% (n=158) are doing residency training and 12.7% (n=162) some post-graduation course. Among the residents, 32.7% (n = 51) were in their first year of training. The five most cited specialties were: 13%, clinical medicine (n = 198); 10%, pediatrics (n = 156); 8%, occupational medicine (n = 122); 7%, gynecology and obstetrics (n = 101); and 6%, general surgery (n = 96). Only 16.4% (n = 210) had no specialty.

**Figure 1:**
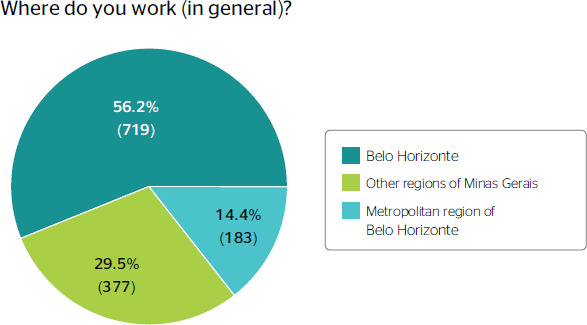
Distribution according to the region where the professionals work.

**Figure 2:**
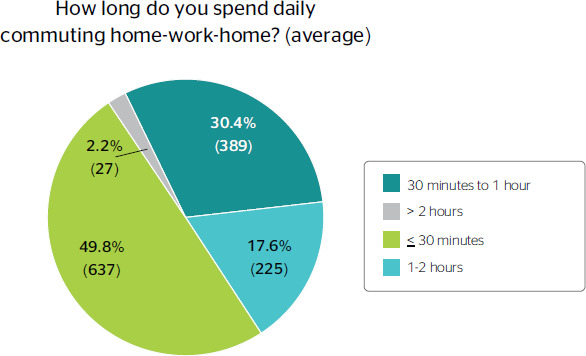
Mean commuting time of professionals on a daily basis.

In relation to the location of work, 56.2% (n = 719) worked in Belo Horizonte, the capital city of the state of Minas Gerais (Figure 1), and 49.8% (n = 638) spent an average of 30 minutes commuting to work (Figure 2).

When asked about satisfaction with their profession, 70.7% (n = 905) responded that they were satisfed with the medical profession, while 7.8% (n = 100) ofen thought about leaving it.

**Figure 3:**
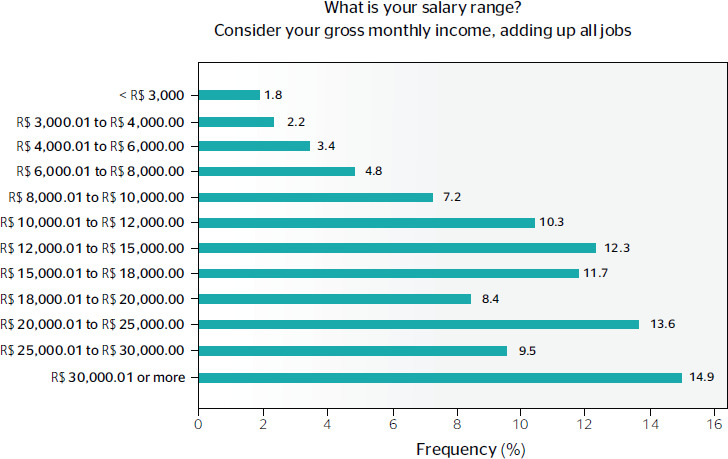
Distribution of the salary range, considering all the workers’ employment bonds.

As for the number of jobs, the population had, on average, 2.56 jobs (SD, 1.46). When asked about feeling overwhelmed, 66.4% (n = 805) stated that they felt overwhelmed. The variation in salary range is presented in Figure 3.

**Figure 4:**
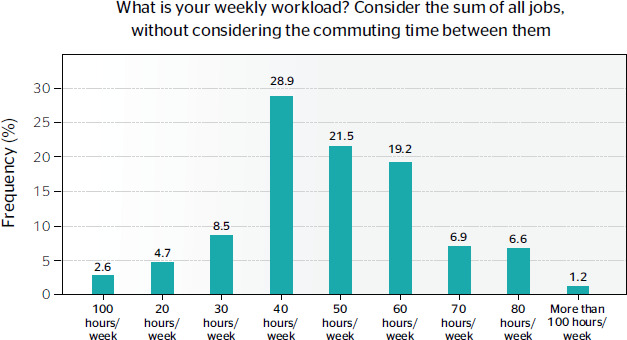
Distribution of weekly workload (%).

Regarding time of from work, 12.9% (n = 165) reported having no days of during the week, and 27.8% (n = 355) had only 1 day of. The distribution of weekly workload is shown in Figure 4.

Regarding satisfaction with their personal life, 73.1% (n = 935) reported feeling satisfed with their personal life. Regarding the ability to socialize, 82.8% (n = 1,080) reported enjoying social interaction activities during leisure time, 75.2% (n = 963) were able to meet with friends outside the medical environment, and 77% (n = 985) considered that they have strong friendship bonds outside the family nucleus. However, 57.4% (n = 735) reported that the medical career prevents or hinders the cultivation of friendships, 82.1% (n = 1,040) have already lost friendship bonds due to lack of time for meetings, 78.3% (n = 1,001) want to expand the opportunities for socialization, and at the same time 43.1% (n = 552) do not feel encouraged to leave home on their days of.

Regarding free time activities, 55.4% (n = 709) do not cultivate any hobby. A choice of several free time activities was given, in which the participant could select more than one activity. The most frequent activities were the following: 67.3% (n = 861) reported staying with the family, 61% (n = 780) reported sleeping and resting without performing other activities, 52.5% (n = 671) performed household activities, 50.1% (n = 640) went to bars and/or restaurants, and 49.5% (n = 634) studied in their spare time.

Concerning spirituality, 71.7% (n = 918) reported following some religion or developing spirituality in some way, with 39% (n = 499) participating in some religious or spiritual activity and 45% (n = 575) having the habit of praying/praying alone.

In the realm of volunteering, 72.3% (n = 925) do not practice any volunteer activity. As for helping some cause, 74.2% (n = 949) contribute with donations, either frequently or not.

About the physical and mental health profile, 60.7% (n = 776) use some medication either by self-prescription or by indication of a colleague, 21.1% (n = 270) are being monitored by a psychologist, 13.8% (n = 176) are being monitored by a psychiatrist, and 38.1% (n = 363) are not being monitored either psychologically or psychiatrically, but consider that they should start some therapy.

Absences reported by the National Institute of Social Security (INSS) or other health insurances related to treating psychiatric conditions occurred in 10.5% (n = 135) of the population investigated.

In relation to clinical diagnoses and treatment of chronic diseases, the study population presented, on average, 1.29 diagnoses (SD, 1.15). The most frequent ones were: 42.7% (n = 545) anxiety; 30.3% (n = 387) depression; 17.4% (n = 222) hypertension; 17.1% (n = 218) obesity; and 10.5% (n = 135) thyroid diseases.

Only 15.2% (n = 193) of the participants reside alone; 58.7% (n = 750) have children, with an average number of children of 1.90 (SD, 0.8), and among those who had children, 61.2% (n = 473) considered that they can have quality time with their children, but less than they would like.

Regarding lifestyle habits, 60.7% (n = 776) use alcoholic beverages and consider that this use does not pose any risk to their health. Of the participants, 5.1% (n = 65) said they smoked, and 0.9% (n = 11) reported using illicit drugs, while 79.8% (n = 1,020) said they did not depend on any substance (legal or not) to relieve the tensions of daily life.

Data on physical activity showed that 30.3% (n = 385) do not practice regular and systematic physical activity, 7.1% do it once a week, 25% twice a week, 32% between three and four times a week, and 5.6%, daily.

About the quality-of-life score indicated by the WHOQOL-Bref, the mean total score in the population was 60.44 (± 21.72). When considering the mean score of each domain, we verified 69.65 (± 16.58) for the physical domain, 60.81 (± 21.35) for the social domain, and 66.77 (± 14.84) for the environmental domain (Table 2). It was possible to stratify the score distribution into three ranges, in which 24.1% (n = 309) had a score below 50, 59.9% (n = 767) had a score greater than 50 and less than 75, and, finally, 15.2% (n = 195) had a score greater than 75, i.e., the group with the highest score.

**Table 2:** Global and domain scores of the World Health Organization Quality of Life instrument-Abbreviated version (WHOQOL-Bref)

Domain	Score		
	Average	SD	Median
Physical	69.65	16.58	71.43
Psychological	64.11	1 7.4 7	66.67
Social	60.81	21.35	66.67
Environment	66.77	14.84	68.75
Global score	60.44	21.72	62.50

SD = standard deviation.

It was observed that the group with individuals that presented the lowest score, suggestive of a lower quality of life score, was mainly composed of younger doctors, at the beginning of their careers, who had long working hours, performed fewer leisure activities, and showed some degree of dissatisfaction with their profession (51.5%), either with their specialty (11.7%) or with medicine as a whole (39.8%).

Also, 89% of the individuals who obtained a mean score below 50 (n = 274) reported feeling overwhelmed, and 57% did not feel fulfilled in their personal life. All these variables were tested by the Mann-Whitney test, with a p < 0.01. Individuals who were married with children showed a higher score in all domains, with a p-value < 0.01 by the Kruskal-Wallis test.

The results of Spearman’s test (Table 3) showed that the number of employment bonds in the different contractual modalities and workplaces did not influence the score in any domain. On the other hand, the salary range, the workload, the commuting time, and the fact of being in medical residency training had a negative impact on the score. Regarding the workload, the higher this variable, the lower the quality-of- life scores. In the tests cited in which there was a positive or negative association, p-value was < 0.01.

**Table 3:** Coefficients of association by Spearman test and statistical significance with the global and domain scores of the World Health Organization Quality of Life instrument-Abbreviated version (WHOQOL-Bref)

Variable	Measure	Domain				
		Physical	Psychological	Social	Environment	Global
Number of leisure activities	Correlation coefficient	0.361	0.397	0.393	0.354	0,379
	p-Value	0.000	0.000	0.000	0.000	0,000
Number of diagnoses	Correlation coefficient	0.427	-0.362	-0.253	-0.231	-0,338
	p-Value	0.000	0.000	0.000	0.000	0,000
Number of employments (types of contract)	Correlation coefficient	-0.015	-0.019	-0.041	0.025	-0,079
	p-Value	0.604	0.493	0.143	0.379	0,005
Has children	Correlation coefficient	0.111	0,161	0.082	0.1 52	0,135
	p-Value	0.002	0.000	0.024	0.000	0,000
Salary range	Correlation coefficient	0.244	0.296	0.190	0.326	0,180
	p-Value	0.000	0.000	0.000	0.000	0,000
Weekly working hours	Correlation coefficient	-0.163	-0.192	-0.218	-0.227	-0,310
	p-Value	0.000	0.000	0.000	0.000	0,000
Number of workplaces	Correlation coefficient	-0.015	-0.002	-0.007	0.010	-0,113
	p-Value	0.587	0.952	0.805	0.731	0,000
Does voluntary work	Correlation oefficient	0.074	0.158	0.129	0.090	0,125
	p-Value	0.008	0.000	0.000	0.001	0,000
Has regular time off	Correlation coefficient	0.183	0.207	0.179	0.239	0,215
	p-Value	0.000	0.000	0.000	0.000	0,000
Practices physical exercises regularly	Correlation coefficient	0.341	0.337	0.279	0.295	0,389
	p-Value	0.000	0.000	0.000	0.000	0,000
Commute time home-work-home	Correlation coefficient	- 0.137	-0.094	-0.092	-0.172	-0,160
	p-Value	0.000	0.001	0.001	0.000	0,000
Age	Correlation coefficient	0.187^*^	0.316	0.213	0.281	0,235
	p-Value	0.000	0.000	0.000	0.000	0,000
Time of experience	Correlation coefficient	0.216^*^	0.342^*^	0.240^*^	0.322^*^	0,272
	p-Value	0.000	0.000	0.000	0.000	0,000

As for lifestyle and health habits, a beter score was observed among those who had regular time of, did some voluntary activity, practiced regular physical activity, and had no medical diagnoses. The p-value observed was < 0.01.

## DISCUSSION

There was no instrument suitable for cross-cultural application to study quality of life until the WHO published the WHOQOL-Bref. A study developed by Fleck et al.^11^ to evaluate the reliability of the WHOQOL-Bref questionnaire concluded that it is a suitable instrument for this purpose, which was confirmed in our research. When applying Cronbach’s test, we obtained similar results to those of the study responsible for validating the questionnaire for the Portuguese version.^11^

Recent publications showed a high rate of conditions compatible with burnout syndrome among medical professionals. A study with 250 American physicians in California conducted by Trockelet al.^12^ applied the Physician Fulfillment Index, the Maslach burnout inventory, and the WHOQOL-Bref to identify the impact of professional fulfillment and the presence of burnout on the quality of life of physicians, indicating that professionals with burnout syndrome and low professional fulfillment had expressively low indices in the quality-of-life scores. Although it was not the objective of this study to diagnose this clinical condition, a high rate of physicians reported feeling overwhelmed, with lower quality-of-life scores among younger professionals, especially among first-year residents, corroborating the findings of Pastura et al.,^13^ which classified the population of resident physicians as more vulnerable to this type of clinical diagnosis.^12^

These authors draw atention to the tendency of physicians to have a negative relationship with work due to an exhaustive working schedule, with high responsibility and lack of resources or inadequacy of those available. Regarding the working day, they verified the high workload’s negative impact on the professional’s quality of life. The study by Abreu-Reis et al.^14^ emphasizes that the average duration of the workload of residents ofen exceeds the 60 hours per week established by law and that there are impacts on the quality of life of physicians when the workload of medical residency is reduced.^13^

The relationship between a heavy workload and low scores in the WHOQOL-Bref is also confirmed in a study conducted in Nigeria (n = 390) by Ogunsunji et al.,^15^ in which physicians with workload equal to or greater than 70 hours per week (primarily residents) scored lower mainly in the physical domain. The same study observed a higher quality of life score among men, specialized professionals, and those over 35 years old. The relationship between age and quality of life is also pointed out among Tai physicians in a study developed by Vutyavanich et al.,^16^ with a sample of 713 physicians from different regions of the country, which indicated that professionals above 50 years old had a beter overall score in the WHOQOL-Bref in comparison to other age groups. However, a different patern was verified in the study by Ghazanfar et al.,^17^ including 1,154 physicians from a province in India, which identified a worse score among older physicians, especially in the psychosocial domain, contradicting the other findings.^15,16,17^

The results found by Abreu-Reis et al.^14^ afer the administration of the WHOQOL-Bref among residents of the same service revealed a worse score in the psychological domain in residents in the first year of residency, with an improvement of the score as the years progressed. In this study, the association of the residency period with the WHOQOL-Bref score was also verified, noting that residents in more advanced stages scored beter than their colleagues in the initial phase of training in all domains, especially in the physical and psychological domains.^14^

Olivares et al.^9^ conducted a similar study among physicians in primary health units in the state of Roraima with 62 participants, observing a high work dissatisfaction rate of 80.6%. The study pointed out the lack of structure and the scarcity of resources and medications as the leading cause of dissatisfaction among professionals in the North region.^9^ It was also observed that physicians who were married, had children, or lived with their families had a better quality-of-life score (p < 0.01). However, no significant difference in scores between sexes was identified, differently from our study, where women had a lower overall mean score compared to men. Our results may be due to the growing trend of feminization of medicine since 2009 and the imposition of a double or triple shift for women physicians, who need to divide time between work responsibilities and household and motherhood demands, as observed by Dias^18^ in 2015. Another study applying the WHOQOL-Bref among residents of a teaching hospital with 84 participants, conducted by Dias et al.^19^ in 2016, found a similar result regarding the association of women with lower scores, as in the study by Ghazanfar et al.,^17^ which also showed a lower score among Indian female physicians.

Regarding the medical diagnoses reported by the participants, the high incidence of reports of anxiety and depression was noteworthy. Depression accounted for 30.3% (n = 387) of the reported diagnoses, representing an incidence about three times higher than the general population, with a prevalence between 4 and 10%.^20,21,22^

According to Botega et al.,^23^ one in every 20 people will be affected by a moderate or severe depressive episode sometime in their lives. The authors also point out that 50% of the people with a depressive episode will develop a second one; of these, 70 to 80% tend to have a third, evidencing that the number of previous episodes is a significant risk factor for the development of new episodes. Given the data described in the present study, it is possible to infer episodes of depression in one out of every three physicians from Minas Gerais.^21,22,23^

According to the WHO, in 2000, depression was the leading cause of disability in the world, evaluated by the index of years lived with disability and considered the fourth largest cause of lost production days. The WHO predicted that by 2020 depression would be the second leading cause of lost workdays due to illness in the world and that by 2030 it will be the world’s most costly non-community illness.^21,22,23^

Manifestations of anxiety can be considered a condition inherent to human beings and a preparation mechanism for situations of threat and danger; however, when present in excess or imbalance, they can lead to the development of anxiety disorders. These include specific, social, and nonspecific phobias, panic disorders, and generalized anxiety disorder.^22,23,24^

In the global scenario, the incidence of anxiety disorders ranges from 0.4 to 3.6%. In the Brazilian context, the prevalence is 9.3% of the population.^22,24^ In the present study, 42.7% (n = 545) of participants reported having been diagnosed with an anxiety disorder, exceeding both global and Brazilian rates, since two in every five participants reported anxiety-related symptoms. This finding is associated with a lower mean score on the WHOQOL-Bref, especially in the psychological and social domains, with p < 0.01. This same association was observed among those who reported a diagnosis of depression, with a p-value < 0.01.

The importance of emotional health in quality of life was also demonstrated in a study developed among 199 physicians from Botucatu, state of São Paulo, which used a questionnaire to assess conscious self-defense mechanisms and compared the results with the WHOQOL-Bref score. The study showed that the volunteers who scored beter in the social and psychological domains had a mature profile of defenses (in the mature profile, characteristics such as sublimation, suppression, rationalization, humor, and anticipation are expected), while physicians with immature defenses had a lower score in all domains. Moreover, low scores on the WHOQOL-Bref were also related to pathological profiles, including alienation, egocentrism, and insecurity characteristics.^25^

Compared to other studies available in the literature, this study presented an expressive and representative sample of the population of physicians in the state of Minas Gerais. The database will allow for other studies to be published on the theme, studying the variables that were not used in this initial analysis.

## CONCLUSIONS

The overall assessment of quality of life using the WHOQOL-Bref among physicians from Minas Gerais does not show alarming levels of low scores. On the contrary, there is a regular score, which would not be considered low if analyzed in isolation. However, when assessing the influencing factors on this population in isolated groups, it is possible to identify that younger physicians, especially women, in their first year of residency, with lower salary ranges, with high workload, without regular time of from work, without opportunities for socialization, and who do not perform leisure activities or physical exercises regularly report more diagnoses of depression and/or anxiety.

Understanding the profile of low quality of life in the medical field will allow us to initiate discussions, propose changes in the medical training process, and develop new studies to suggest practical actions that positively impact these professionals’ quality of life. Based on these epidemiological data, the objective was to guide initiatives to reduce the alarming and growing levels of depression, anxiety, burnout syndrome risk, and suicide in this population, especially at the beginning of their careers, so that there is an environment of well-being in all phases of a physician’s life, from graduation to retirement.
